# Integration of Agronomic Practices with Herbicides for Sustainable Weed Management in Aerobic Rice

**DOI:** 10.1155/2013/916408

**Published:** 2013-10-02

**Authors:** M. P. Anwar, A. S. Juraimi, M. T. M. Mohamed, M. K. Uddin, B. Samedani, A. Puteh, Azmi Man

**Affiliations:** ^1^Institute of Tropical Agriculture, Universiti Putra Malaysia, 43400 Serdang, Selangor, Malaysia; ^2^Department of Agronomy, Bangladesh Agricultural University, Mymensingh 2202, Bangladesh; ^3^Department of Crop Science, Universiti Putra Malaysia, 43400 Serdang, Selangor, Malaysia; ^4^Malaysian Agricultural Research and Development Institute, 50774 Kuala Lumpur, Malaysia

## Abstract

Till now, herbicide seems to be a cost effective tool from an agronomic view point to control weeds. But long term efficacy and sustainability issues are the driving forces behind the reconsideration of herbicide dependent weed management strategy in rice. This demands reappearance of physical and cultural management options combined with judicious herbicide application in a more comprehensive and integrated way. Keeping those in mind, some agronomic tools along with different manual weeding and herbicides combinations were evaluated for their weed control efficacy in rice under aerobic soil conditions. Combination of competitive variety, higher seeding rate, and seed priming resulted in more competitive cropping system in favor of rice, which was reflected in lower weed pressure, higher weed control efficiency, and better yield. Most of the herbicides exhibited excellent weed control efficiency. Treatments comprising only herbicides required less cost involvement but produced higher net benefit. On the contrary, treatments comprising both herbicide and manual weeding required high cost involvement and thus produced lower net benefit. Therefore, adoption of competitive rice variety, higher seed rate, and seed priming along with spraying different early-postemergence herbicides in rotation at 10 days after seeding (DAS) followed by a manual weeding at 30 DAS may be recommended from sustainability view point.

## 1. Introduction

Weeds are endemic in crops [1] and a constant problem in crop production because of their dynamic nature [2]. Despite modern control practices aimed at weed elimination, weed continues to be a ubiquitous and recurrent threat for crop production due to its ability to shift in response to management practices and environmental conditions [3]. Because of the diversity and plasticity of weed communities, weed management should include diverse approaches and to be viewed as a continuous process [2]. Physical, cultural, and biological weed management was the only weed control strategy till 1940s. Since the introduction of herbicides, their amazing performance led to the belief that herbicide would solve the weed problem forever. But concern over the escalating problems of herbicide persistence and resistance in weeds and herbicide toxicity to crop has reinforced the need for alternative approaches [1]. Herbicides are often blamed for environmental pollution [4] and impoverishment of the natural flora and fauna in agro ecosystem [5]. Long term efficacy and sustainability issues are also the driving forces behind the reconsideration of herbicide dependent weed management.

In response to aforesaid problems, rice farming has been challenged to adopt a weed management strategy more respectful for environment. Weed management continues to be a huge challenge in aerobic rice which is highly vulnerable to weed infestation because of dry ploughing and aerobic soil conditions [6]. Proper weed management is considered to be one of the most important prerequisites to ensure satisfactory yield of rice [7, 8]. High weed pressure in direct seeded rice lowers the economic return, and in extreme cases rice cultivation results in a losing concern [9]. This demands reappearance of physical, cultural, and biological weed management combined with judicious application of herbicides based on a thorough understanding in the crop-weed ecology, known as integrated weed management (IWM). The IWM is a component of integrated pest management which involves the integration of effective, environmentally safe, and socially acceptable control tactics that reduce weed interference below the economic injury level [10–13]. The IWM has got the potential to reduce herbicide use and to provide a robust and sustainable weed management [14]. The ultimate challenge towards developing an effective IWM is to create a cropping system unfavorable for weeds and favorable for crop [2].

Although weed management is herbicide dominated in many rice belts, there are strong indications that it will change in future [15]. Because farmers are now very much concerned about the advent of herbicide resistance and unwarranted environmental hazard, and therefore are becoming increasingly interested in less herbicide dependent weed management approach [1, 16], many farmers are using IWM approach for controlling weeds, but to some extent are hard to measure [11]. For the less herbicide dependent sustainable rice farming, IWM, has been emphasized by Azmi and Baki [17]. As stated by Jayadeva et al. [18], IWM can be successfully implemented in aerobic rice. None of the control measures in single can provide acceptable levels of weed control, and therefore, various components are to be integrated in a logical sequence [14]. Various agronomic tools like tillage, competitive cultivar, crop rotation, seeding date, seeding density, cover crop, and fertilizer management have been evaluated for their potentiality in managing weeds [19–23]. But all those tools may not work in every situation or with every weed/crop species [1]. Therefore, before integration, each of the components needs to be evaluated for their efficacy.

In rice, many studies have been conducted on IWM, most of which have looked at one or two of those components in isolation. Since the concept of aerobic rice is new, IWM issue is yet to be addressed properly considering the diverse weed management approaches. Therefore, for designing a sustainable weed management strategy for aerobic rice, it is a prerequisite to assess the simultaneous effect of different agronomic practices combined with timely herbicide application. The present study was, therefore, conducted to find out suitable herbicide and manual weeding combination(s) simultaneously incorporated with different agronomic practices to provide a comprehensive integrated weed management system for aerobic rice variety AERON 1.

## 2. Materials and Methods

### 2.1. Experimental Site and Soil

The field trials were conducted in main season 2010/2011 (November–January) and off season 2011 (May–July) at Universiti Putra Malaysia, Malaysia (3°00′ 21.34′′N, 101°42′ 15.06′′E, and 37 m elevation). The experimental soil (Serdang series) was sandy clay loam in texture (56.77% sand, 21.30% silt, and 21.93% clay) and acidic in reaction (pH 5.6) with 1.42 g cc^−1^ bulk density, 1.77% organic carbon, and 17.24 me 100 g^−1^ soil CEC. Soil contained 0.38% total N, 21.5 ppm available P, 139 ppm available K, 803 ppm Ca, and 159 ppm Mg. At field capacity, soil water retention was 22.69% (wet basis) and 29.35% (dry basis). The local climate was hot humid tropic with plentiful rainfall. During the experimental period, average maximum and minimum temperatures, relative humidity, rainfall, evaporation, and sunshine hours ranged from 31.7 to 35.0°C and 22.9 to 24.4°C, 93.5 to 94.7%, 3.8 to 9.9 mm day^−1^, 2.94 to 4.82 mm day^−1^ and 3.95 to 6.97 hrs day^−1^, respectively.

### 2.2. Plant Material

Aerobic rice variety AERON 1 was used as the plant material in the present study. This variety is sourced from International Rice Research Institute (IRRI).

### 2.3. Experimental Treatments and Design

The experiment was laid out in a randomized complete block design with three replications. Fourteen different combinations of herbicides and manual weeding were evaluated for their efficacy in controlling weeds under aerobic soil conditions; season-long weed-free check and season-long weedy check were also included in the trial (Table 1). Herbicides included one preemergence (pretilachlor), five early-postemergence (cyhalofop-butyl, bensulfuron, bispyribac-sodium, propanil and thiobencarb), and two postemergence (bentazon and MCPA), which were available as six commercial products. Herbicides used in this experiment were selected based on their performances in the earlier study [24]. Season-long weed-free plots were maintained through manual weeding as and when necessary. In weedy checks, no weeding operations were done.

### 2.4. Integration of Agronomic Practices

Different agronomic tools were integrated in this study to create a more competitive condition in favor of rice and hence to achieve higher weed control efficiency. Aerobic rice variety AERON 1 was used as the plant material, since it was the most competitive and productive weed under aerobic soil conditions as found in the previous study [21]. A seeding rate of 300 seeds/m^2^ was used for better weed competitiveness and higher yield as evident in the earlier study [22]. Based on the findings of the preceding study [25], rice seeds were primed by soaking in 1% Zappa solution for 24 hours followed by air drying for 12 hours to boost weed competitiveness through faster and higher emergence rate and increased seedling vigor. Timing of herbicide application and manual weeding was adjusted to match with the predetermined critical period of weed control of 20–43 days after seeding [26] at 10% yield loss level.

### 2.5. Crop Husbandry

The soil was dry-ploughed and harrowed but not puddled during preparation. Rice seeds were directly dry-seeded at 2 cm depth in rows with 25 cm interrow and 15 cm intrarow spacing at the rate of 300 seeds/m^2^. Each plot, of size 5 m long and 3 m wide, was fertilized with triple super phosphate (TSP) and muriate of potash (MP) at the rate of 100 kg P/ha and 100 kg K/ha, respectively, during final land preparation; urea was top dressed thrice each at the rate of 50 kg N/ha at 2, 4, and 6 weeks after seeding. Soil was maintained under nonsaturated aerobic conditions throughout. The trial was primarily rain fed, but supplemental sprinkler irrigation was given when hair-like cracks appeared on the soil surface. Overflow canals were kept to facilitate drainage following heavy rainfall to avoid ponding. Different intercultural operations and plant protection measures were taken following standard practices [27].

### 2.6. Weed Measurements

A 25 cm × 25 cm quadrate was randomly placed lengthwise at four spots in each plot for recording of weed data at 10, 30, and 75 days after seeding (DAS). Weeds were clipped to ground level, identified and counted by species, and separately oven dried at 70°C for 72 h. Weed density (WD) and weed dry weight (WDW) were expressed as no./m^2^ and g/m^2^, respectively. Dominant weed species were identified using the summed dominance ratio (SDR) computed as follows [28]:

(1) SDR of a weed species =[Relative density (RD)+Relative dry weight (RDW)]2,

where

(2) RD (%)=(Density of a given weed speciesTotal weed density)×100,RDW (%)=(Dry weight of a given weed speciesTotal weed dry weight)×100.

Weed control rating was done visually at 7, 14, and 21 days after herbicide application (DAA) of each herbicide using a scale of 1 to 5 [29]. Weed control efficiency (WCE) of different herbicide treatments was calculated as follows [30]:

(3) WCE (%)=(DWC – DWT)DWC×100,

where

(4) DWC=dry weight of weeds in weedy check plots,DWT=dry weight of weeds in treated plots.

### 2.7. Rice Measurements

At maturity, yield attributes were recorded from ten randomly selected hills. All the panicles of sample hills were counted and converted to panicles/m^2^. Sample panicles were hand threshed; filled grains were separated from unfilled grains and counted to calculate filled grains/panicle. Central 3 m^2^ area of each plot was hand harvested to record grain yield (t/ha) and thousand-seed weight (g). Grain yield and thousand-seed weight were adjusted to 14% moisture content. Percent relative yield loss (RYL) due to weeds was calculated as [100 × (weed-free yield − weedy yield)/weed-free yield]. Crop phytotoxicity rating of different herbicides was assessed visually at 7, 14, and 21 days after application (DAA) of herbicide using a scale of 1 to 5 [29].

### 2.8. Economic Measurements

Economic analysis was performed following the procedure by Hussain et al. [31]. Two manual weedings were considered sufficient to keep the plots weed-free throughout. Laborers required for one round weeding and one round herbicide spraying per hectare were 50 and 2, respectively. The cost for laborer was Ringgit Malaysia (RM) 25/laborer/day. The cost of each herbicide was estimated based on their local market price. Price of paddy was collected from different rice growing areas and was considered as RM 1000/t for calculating the gross return. The net benefit per hectare for each treatment was calculated by deducting the weed management cost from the gross return.

### 2.9. Statistical Analysis

All data were subjected to ANOVA by using SAS statistical software package version 9.1 [32]. Since treatment by season interaction was not significant, data were averaged across the seasons and were used in subsequent analysis. Significant differences among means were adjudged by using Fisher's protected least significant difference (LSD) test at *P* ≤ 0.05. Simple regression analysis was conducted to quantify the relationship among different traits.

## 3. Results

### 3.1. Composition of Weed Flora

The study was conducted under naturally occurring mixed weed population. The experimental field was infested with broadleaf weeds, sedges, and grasses, and mostly dominated by broadleaf weeds. The weed community had a wide spectrum of 19 species representing 11 different families (Table 2). Among those, 17 species were prevalent in both main and off seasons. Further analysis showed that the relative composition of the broadleaf, sedges, and grasses were about 62, 19, and 19%, respectively, in main season, while the respective values in off season were 55%, 23%, and 22%.

### 3.2. Weed Control and Crop Toxicity Ratings

Weed control was rated visually at 7, 14, and 21 DAA of herbicide (Table 3). The rating showed that application of preemergence herbicide pretilachlor/safener resulted in excellent control of weeds at 7 DAA, but good and fair control at 14 and 21 DAA, respectively. On the other hand, early postemergence application of cyhalofop-butyl + bensulfuron and bispyribac-sodium provided excellent weed control at 21 DAA, although the ratings were fair at 7 DAA and good at 14 DAA. Spraying with propanil/thiobencarb offered moderate weed control at 7 DAA but good control both at 14 and 21 DAA. The only postemergence formulation bentazon/MCPA resulted in poor control at 7 DAA, fair control at 14 DAA, and excellent control at 21 DAA. Thus, weed control rating varied with course of time and herbicide formulation. In general, all the herbicides showed high selectivity to rice crop (Table 3). Among the herbicides, propanil/thiobencarb and cyhalofop-butyl + bensulfuron caused no visible injury to rice plant, while pretilachlor/safener, bispyribac-sodium, and bentazon/MCPA showed slight phytotoxicity to rice. hytotoxicity of pretilachlor/safener was characterized by minor reduction in plant height and slight leaf chlorosis as observed at 7 DAA, while in case of Bispyribac-sodium and bentazon/MCPA, plant growth was stunted to some extent, and leaves failed to expand fully and became yellowish as noticed at 7 and 14 DAA. However, those symptoms could not persist up to crop harvest.

### 3.3. Weed Dry Weight and Density

Both weed dry weight and density were significantly influenced by weed control treatments at all the sampling dates (Table 4). At 10 DAS, that is, just before application of early-postemergence herbicides, only the plots sprayed with pretilachlor/safener produced lower weed dry weight and density compared to season-long weedy check. On an average, weed dry weight and density in pretilachlor/safener treated plots were 2 g/m^2^ and 52 weeds/m^2^, respectively, while for weedy check, the respective values were >6 g/m^2^ and 166 weeds/m^2^. Hence, preemergence application of pretilachlor/safener reduced weed dry weight by 70% and weed density by 69% as compared to untreated weedy plots. Other weed control treatments comprising only early-post- with/without postemergence herbicides or manual weeding produced similar weed dry weight and density with season-long weedy check. Significant effect of different early-postemergence herbicides on weed dry weight and density was evident from the observations recorded at 30 DAS (prior to application of postemergence herbicide bentazon/MCPA or manual weeding) (Table 4). Reduction in weed dry weight and density due to application of different early-postemergence herbicides ranged from 40 to 90% and 39 to 90%, respectively. Spraying with propanil/thiobencarb after pretilachlor/safener resulted in highest reduction (90%) in weed dry weight and density, while the plots treated with Pretilachlor/safener registered the highest weed dry weight and density next to untreated weedy plots. The early-postemergence herbicides performed in the order of cyhalofop-butyl + bensulfuron > propanil/thiobencarb > bispyribac-sodium in terms of weed dry weight reduction. Weed control treatments exerted significant influence on weed dry weight and density at 75 DAS (Table 4). Most of the treatments provided excellent weed control while others performed satisfactorily. Weed dry weight in different weed control treatments ranged between 6.77 and 64.58 g/m^2^ and density between 76.77 and 134 plants/m^2^, while in weedy check the respective values were 328.51 g/m^2^ and 299.50 plants/m^2^. Results showed that early-postemergence application of any of the herbicides at 10 DAS followed by a manual weeding at 30 DAS or preemergence application of pretilachlor/safener at 1 DAS followed by early-postemergence application of propanil/thiobencarb at 10 DAS followed by manual weeding/postemergence application of bentazon/MCPA at 30 DAS resulted in the lowest and identical weed dry weight and density. This negates the necessity of preemergence application. It is interesting to note that postemergence application of bentazon/MCPA at 30 DAS and manual weeding on the same day resulted in similar weed dry weight and density reduction irrespective of early-postemergence application. But bentazon/MCPA was found less effective than manual weeding when applied after preemergence application of pretilachlor/safener not followed by an early-postemergence application.

### 3.4. Weed Control Efficiency

**Figure 1:**
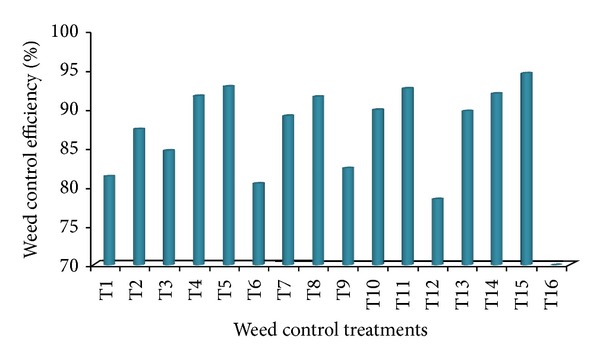
Weed control efficiency of different weed control treatments in aerobic rice variety AERON 1 (averaged over seasons). T1: pretilachlor/safener fb bentazon/MCPA; T2: pretilachlor/safener fb manual weeding; T3: pretilachlor/safener fb propanil/thiobencarb; T4: pretilachlor/safener fb propanil/thiobencarb fb bentazon/MCPA; T5: pretilachlor/safener fb propanil/thiobencarb fb manual weeding; T6: propanil/thiobencarb; T7: propanil/thiobencarb fb bentazon/MCPA; T8: propanil/thiobencarb fb manual weeding; T9: cyhalofop-butyl + bensulfuron; T10: cyhalofop-butyl + bensulfuron fb bentazon/MCPA; T11: cyhalofop-butyl + bensulfuron fb manual weeding; T12: bispyribac-sodium; T13: bispyribac-sodium fb bentazon/MCPA; T14: bispyribac-sodium fb manual weeding; T15: season long weed-free by manual weeding; T16: season long weedy.

The WCE based on the weed dry weight at harvest varied significantly among the weed control treatments (Figure 1). All the weed control treatments showed more than 80% WCE, and some treatments performed similar to season-long weed-free check with almost 98% WCE. It is evident that pretilachlor/safener fb propanil/thiobencarb fb manual weeding resulted in the highest WCE (97.93%) identically followed by cyhalofop-butyl + bensulfuron fb manual weeding (97.60%), bispyribac-sodium fb manual weeding (96.85%), pretilachlor/safener fb propanil/thiobencarb fb bentazon/MCPA (96.45%), and propanil/thiobencarb fb manual weeding (96.35%) (Figure 1). Bispyribac-sodium, on the other hand, showed the lowest WCE (80.34%) closely followed by propanil/thiobencarb (82.75%), pretilachlor/safener fb bentazon/MCPA (83.90%), and cyhalofop-butyl + bensulfuron (85.16%). The present findings confirm that early-postemergence application of any of the herbicides under study at 10 DAS followed by manual weeding/postemergence application of Bentazon/MCPA at 30 DAS would result in excellent weed control.

### 3.5. Yield and Yield Attributes

Grain yield of AERON 1 varied significantly due to weed control treatments (Table 5). All the treatments resulted in significantly higher yield than weedy check did, and several treatments generated yield as high as weed-free yield. Pretilachlor/safener fb propanil/thiobencarb fb bentazon/MCPA or manual weeding, propanil/thiobencarb fb bentazon/MCPA or manual weeding, cyhalofop-butyl + bensulfuron fb bentazon/MCPA or manual weeding, and bispyribac-sodium fb manual weeding performed excellent in terms of yield (ranging from 4.40 to 4.55 t/ha) which were statistically similar to that obtained from weed-free check (4.68 t/ha). pretilachlor/safener fb propanil/thiobencarb or fb bentazon/MCPA or manual weeding and bispyribac-sodium fb bentazon/MCPA also recorded acceptable yield (>4 t/ha), very close to weed-free yield. Propanil/thiobencarb, cyhalofop-butyl + bensulfuron, and bispyribac-sodium, on the contrary, recorded comparatively lower yield (ranging between 3.66 and 3.77 t/ha) but still much higher than weedy yield (1.77 t/ha). Yield attributes were significantly affected by herbicide treatments (Table 5). All the attributes attained their highest values in weed-free check and lowest values in the weedy check. In general, pretilachlor/safener fb propanil/thiobencarb fb bentazon/MCPA or manual weeding, propanil/thiobencarb fb bentazon/MCPA or manual weeding, cyhalofop-butyl + bensulfuron fb bentazon/MCPA or manual weeding, and bispyribac-sodium fb bentazon/MCPA or manual weeding performed best in terms of yield attributes. Single application of propanil/thiobencarb or cyhalofop-butyl + bensulfuron or bispyribac-sodium and application of propanil/thiobencarb fb bentazon/MCPA or manual weeding or propanil/thiobencarb resulted in the poorest yield attributes.

### 3.6. Relative Yield Loss

Relative yield loss due to weed varied widely (2.78–23.081%) among the weed control treatments (Table 5). In weedy check, RYL was recorded as high as 62.18%. Pretilachlor/safener fb propanil/thiobencarb fb manual weeding allowed the least yield penalty of only 2.78% closely followed by cyhalofop-butyl + bensulfuron fb manual weeding (3.21%), pretilachlor/safener fb propanil/thiobencarb fb bentazon/MCPA (4.06%), cyhalofop-butyl + bensulfuron fb bentazon/MCPA (4.49%), and bispyribac-sodium fb manual weeding (4.91%). A single application of bispyribac-sodium or propanil/thiobencarb or cyhalofop-butyl + bensulfuron resulted in high RYL (>20%). The remaining treatments allowed moderate RYL ranging between 6 and 13%. 

### 3.7. Economics

Weed control treatments showed a wide range of economic return (Table 6). Cost analysis revealed that the highest net benefit of Ringgit Malaysia (RM) 4086/ha was recorded with cyhalofop-butyl + bensulfuron fb bentazon/MCPA closely followed by bispyribac-sodium fb bentazon/MCPA (RM 4080/ha), propanil/thiobencarb fb bentazon/MCPA (RM 3988/ha), and pretilachlor/safener fb propanil/thiobencarb fb bentazon/MCPA (RM 3911/ha). Pretilachlor/safener fb bentazon/MCPA and pretilachlor/safener fb propanil/thiobencarb also resulted in high net benefit (RM 3773/ha and RM 3761/ha, resp.). A single early-postemergence spray with propanil/thiobencarb or bispyribac-sodium or cyhalofop-butyl + bensulfuron recorded moderate net benefit ranging from RM 3368/ha to RM 3496/ha. On the other hand, when manual weeding was integrated with herbicides, net benefits were found lower (ranging from RM 2713/ha to RM 3030/ha) as compared with those obtained from only herbicide application. The season-long weed-free plots resulted in a net benefit of only RM 2180/ha which was not much higher than that obtained from season-long weedy plots (RM 1770/ha), and comparatively lower than that of any of the treatments. Despite the highest gross income (RM 4680/ha), season-long weed-free plots resulted in very low net benefit because of high cost involvement in manual weeding (RM 2500/ha). The results further revealed that in every case when bentazon/MCPA was replaced by manual weeding, gross income was increased marginally but net benefit was reduced considerably because of much higher cost involvement in manual weeding (RM 1250/ha) compared to Bentazon/MCPA (RM 110/ha). Thus, integrated weed management resulted in lower economic return compared to herbicide based management.

## 4. Discussion

The existence and risk of developing herbicide resistance and concern about herbicidal impact on environment and public health make herbicide dominated weed management increasingly vulnerable. To reduce herbicide reliance, one approach is to adjust crop management practices such that crop-weed interactions are altered to the benefit of the crop [15], but this is not enough to manage weeds. Till now herbicide is a cost effective tool to fight against weeds, and therefore, weed management system using herbicides probably will continue. The present study advocates an integrated approach of weed management for aerobic rice with a reduced reliance on herbicides.

Naturally occurring weed flora of the study area represented 19 weed species belonging to 11 families. Broadleaf was the most dominant group followed by sedges and grasses. Weed community in the aerobic rice is generally dominated by broadleaf weeds followed by sedges and grasses [18, 33]. Anwar et al. [24] also reported that the relative composition of the broadleaf, sedges, and grasses were about 60%, 20%, and 20%, respectively, in aerobic rice field, and the most dominant weed species were *P. heterophylla* Nees, *S. dulcis, C. rutidosperma,* and *C. rotundus*. In contrast, Jaya Suria et al. [34] accounted from their trial with aerobic rice that grassy weeds constituted about 80% of total weed community. Greater abundance of broadleaf weeds under saturated conditions and dominance of sedges, and grasses under dry seeded/aerobic conditions have also been documented by Moody and Drost [35]. The differences in the weed composition might be due to variation in agroecological conditions, management practices, and weed seed bank composition among the study areas.

Across the seasons, weed dry weight and density in the weedy check were recorded as 328 g/m^2^ and 299/m^2^, respectively. The high weed pressure under aerobic soil conditions as observed in this study is long established as reported by many researchers [24, 25, 33, 36, 37]. Dry tillage coupled with aerobic soil conditions [6], lack of a “head start” of rice seeds over germinating weed seeds [38], and absence of flooding to suppress the initial flush of weeds [39] are the plausible causes behind the high weed invasion in aerobic rice. As De Datta and Baltazar [40] stated, rice ecosystem and management practices mostly determine weed pressure, rice-weed competition, and ultimately the weed control tactics. Therefore, understanding the weed community in terms of species dominance pattern and pressure is necessary for successful weed management. The weed density and dry weight recorded in this study are, respectively, 36 and 14% lower than those obtained from herbicide screening trial [24] and 39 and 20% lower than those observed in critical period study [26]. All the three studies were conducted with rice germplsm AERON 1 in the same seasons at the same site and set of agroclimatic conditions. Despite the likeness in spatiotemporal aspects among those three studies, much lower weed pressure in the present study as compared to those of previous studies might be the contribution of integrating higher seed rate (300 instead of 200 seeds/m^2^) and seed invigoration (Zappa primed seeds instead of unprimed ones). Sowing primed seeds at a higher rate enhanced the competitiveness of rice against weeds which eventually reduced weed density and dry weight.

In this study, 14 different combinations of six commercial herbicide products were integrated with manual weeding aimed at controlling weeds during the predetermined critical period of weed competition of 20–43 days after seeding [26]. Every herbicide formulation was found effective in arresting weed growth. All the weed control treatments showed high weed control efficiency (>80% WCE), and a number of them provided excellent weed control (>95% WCE). Preemergence application fb early-postemergence application fb postemergence application or manual weeding and early-postemergence application fb postemergence application or manual weeding resulted in higher WCE as compared with a single early-postemergence application. This might be due to that late emerged weeds remained uncontrolled when preemergence or early-postemergence application was not followed by a spraying with postemergence herbicide or manual weeding at midgrowth stages of rice to cover the entire critical period of weed competition. These results are in line with that of Sunil et al. [33], who opined that for achieving high WCE in aerobic rice a preemergence application must be followed by a manual weeding at 40 DAS. Highest WCE in aerobic rice variety AERON 1 resulted from early-postemergence application at 10 DAS followed by a manual weeding at 43 DAS has also been reported by Jaya Suria et al. [34]. The WCEs obtained from this study (ranging from 80 to 98%) are comparatively higher than those recorded with the previous study (ranging from 53 to 92%) conducted at the same site considering same herbicides [24]. Combined merits of higher seeding rate and seed priming provided a more competitive cropping system in favor of rice in the present study compared to the previous one, which might enhance herbicide efficacy. Earlier studies have similarly documented that improved agronomic practices can effectively suppress weed growth and increase herbicide efficacy [1, 41].

None of the herbicides under study caused any significant injury to rice plant, and thus, they exhibited high selectivity to rice. Minor injuries were evident with few herbicides which disappeared shortly. At the late season evaluation, no injury was visible from any herbicide treatment. As observed in the earlier study [24], bentazon/MCPA applied at 40 DAS exhibited no injury to rice plant at 7 DAA, while in the present study, when applied at 30 DAS to match with the critical period of weed competition, bentazon/MCPA caused slight phytotoxicity to rice at 7 DAA. A possible explanation for this might be that rice plant is susceptible to bentazon/MCPA if applied before 40 DAS. No phytotoxicity was observed in AERON 1 due to bentazon/MCPA application at 43 DAS [34]. Thus, herbicide phytotoxicity is crop growth stage specific as confirmed by Levene and Owen [42]. Herbicides in this study were applied following manufacturers' recommendations, which might result in no/very slight injury to rice plants. Moreover, aerobic soil conditions helped reduce herbicide injury to the crop [43]. In fact, rice plant shows high tolerance to herbicides and may suffer slight initial injuries which disappear shortly, but seldom phytotoxicity persists up to crop harvest [43].

Weed control efficiency (WCE) was eventually translated into grain yield. All the weed control treatments significantly out-yielded weedy check, and some performed as good as weed-free check because of their high WCE. In contrast, weed control treatments with low WCE resulted in reduced yield. The WCE is also reflected in RYL. As evident from this study, the higher the WCE the lower the RYL. The increase in rice grain yield by increasing WCE has also been reported by others [24, 34]. Reasonably, weedy check allowed the maximum RYL. In fact, weed removal reduces interspecific competition for resources and enables crop to utilize available resources more efficiently than weeds which eventually results in higher yield. Early-postemergence application at 10 DAS followed by postemergence application or manual weeding at 30 DAS resulted in similar grain yield with season long weed-free or preemergence application at 1 DAS followed by early-postemergence application at 10 DAS followed by postemergence application or manual weeding at 30 DAS. On the other hand, a single early-postemergence application at 10 DAS not followed by a postemergence application or manual weeding at 30 DAS resulted in considerable yield reduction compared to weed-free check. This finding justifies the implication of critical period of weed competition and confirms that presence of weeds before or after critical period of weed competition (20–43 DAS for this study) will not pose a threat to crop yield, and yield obtained by keeping weed-free during CPWC is almost similar to that obtained by keeping weed-free throughout [26]. Also, both weedy and weed-free yields were recorded higher and RYL against different weed control treatments were recorded lower in the present study as compared to the earlier study [24]. This result may be explained by the fact that the use of primed seed coupled with higher seed rate in the present study resulted in faster and higher emergence rate along with vigorous stand offering rice plants an advantage to tolerate and outcompete weeds, which is ultimately translated into higher yield. It is encouraging to compare this finding with that of Blackshaw et al. [1] who confirmed that integration of agronomic practices like seeding rate and seeding time in conjunction with limited herbicide use could increase crop yield and reduce weed pressure.

In the present study, all the yield attributes responded significantly to weed control treatments. These findings are in agreement with those of Sunil et al. [33], who observed that all the yield attributes of aerobic rice were significantly influenced by weed control treatments. In contrast, Juraimi et al. [44] found that only number of panicle/m^2^ was influenced by weed control treatments. Thus, responses of yield components of rice to herbicide treatments are variable.

The economics of a weed control method is determined by its cost involvement and WCE. Similarly as Wibawa et al. [45] stated, the economics of herbicide depends on the price, recommended dosage, and efficacy. In this study, cost of different weed control treatments varied widely. Treatments comprising only herbicides required low cost involvement and thus produced higher net benefit, on the contrary, treatments comprising both herbicide and manual weeding required high cost involvement and resulted in lower net benefit. In season-long weed-free check, despite the maximum gross income (RM 4680/ha), net benefit was much lower (RM 2180/ha) and very close to that of weedy check (RM 1770/ha). Season-long weed-free check generated an additional return of only RM 410/ha over season-long weedy checks. When bentazon/MCPA was replaced by manual weeding, gross income increased but net benefit decreased because of high cost involvement in manual weeding. Even a single early-postemergence application generated more net benefit as compared to early-postemergence application followed by manual weeding. Consequently, manual weeding is less remunerative compared to herbicidal control, and practicing manual weeding throughout the season is a losing concern, confirming the view of many others [31, 33, 37]. Compared to earlier study [24], higher gross income and higher net benefit were encountered in the present study, which might be due to better yield and higher WCE as a consequence of more competitive cropping system in favor of rice.

## 5. Conclusions

**Figure 2:**
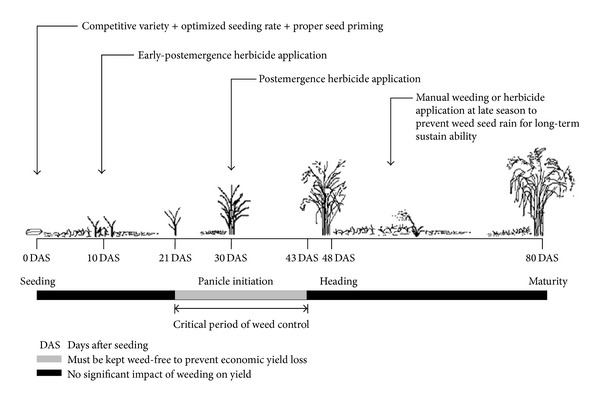
Integrated weed management schedule for aerobic rice production.

A more competitive cropping system in favor of rice as a consequence of combined use of competitive variety, higher seeding rate, and seed priming is evident from the study, which was reflected in lower weed pressure, higher weed control efficiency, and better yield. Weed control only during critical period of competition is also justified as some weed control treatments produced yield similar to weed-free yield. Herbicide and manual weeding combinations resulted in lower net benefit compared to herbicidal control because of high cost involvement in manual weeding. From economic view points, application of cyhalofop-butyl + bensulfuron or bispyribac-sodium or propanil/thiobencarb at 10 DAS followed by bentazon/MCPA at 30 DAS can be recommended, while for the sustainability of long-term weed management, cyhalofop-butyl + bensulfuron or bispyribac-sodium or propanil/thiobencarb should be applied in rotation at 10 DAS followed by a manual weeding at 30 DAS. Based on the findings of this study, an integrated weed management schedule for aerobic rice has been presented in Figure 2.

## Figures and Tables

**Table 1:** List of weed control treatments used in the experiments in main season 2010/11 and off season 2011.

Label	Treatments	Application rate	Time (DAS)
T1	Pretilachlor/safener fb bentazon/MCPA	0.5 kg a.i./ha fb 0.6/0.1 kg a.i./ha	1 fb 30
T2	Pretilachlor/safener fb MW	0.5 kg a.i./ha	10 fb 30
T3	Pretilachlor/safener fb propanil/thiobencarb	0.5 kg a.i./ha fb 1.2/2.4 kg a.i./ha	1 fb 10
T4	Pretilachlor/safener fb propanil/thiobencarb fb bentazon/MCPA	0.5 kg a.i./ha fb 1.2/2.4 kg a.i./ha fb 0.6/0.1 kg a.i./ha	1 fb 10 fb 30
T5	Pretilachlor/safener fb propanil/thiobencarb fb MW	0.5 kg a.i./ha fb 1.2/2.4 kg a.i./ha	1 fb 10 fb 30
T6	Propanil/thiobencarb	1.2/2.4 kg a.i./ha	10
T7	Propanil/thiobencarb fb bentazon/MCPA	1.2/2.4 kg a.i./ha fb. 0.6/0.1 kg a.i./ha	10 fb 30
T8	Propanil/thiobencarb fb MW	1.2/2.4 kg a.i./ha	10 fb 30
T9	Cyhalofop-butyl + bensulfuron	0.1 kg a.i./ha + 0.06 kg a.i/ha	10
T10	Cyhalofop-butyl + bensulfuron fb bentazon/MCPA	0.1 kg a.i./ha + 0.06 kg a.i./ha fb 0.6/0.1 kg a.i./ha	10 fb 30
T11	Cyhalofop-butyl + bensulfuron fb MW	0.1 kg a.i./ha + 0.06 kg a.i./ha	10 fb 30
T12	Bispyribac-sodium	0.03 kg a.i./ha	10
T13	Bispyribac-sodium fb bentazon/MCPA	0.03 kg a.i./ha fb 0.6/0.1 kg a.i./ha	10 fb 30
T14	Bispyribac-sodium fb MW	0.03 kg a.i./ha	10 fb 30
T15	Season long weed-free		Season long
T16	Season long weedy		—

/: means that the herbicides were formulated as a proprietary mixture, +: means that the herbicides were tank-mixed and applied at the same time, all herbicides were applied as per manufacturers' recommended rates in 300 L of water per hectare by knapsack sprayer. DAS: days after seeding; fb: followed by.

**Table 2:** Weed composition with summed dominance ratio (SDR) followed by standard error (SE) in off season 2010 and main season 2010/2011 as observed in season-long weedy check.

Scientific name	Family name	SDR (SE)	
		Main season 2010/2011	Off season 2011
Broadleaves			
*Physalis heterophylla* Nees	Solanaceae	18.36 ± 6.4	15.54 ± 3.78
*Scoparia dulcis* L.	Scrophulariaceae	12.50 ± 4.2	10.32 ± 3.41
*Cleome rutidosperma* DC	Capparidaceae	12.38 ± 3.9	9.30 ± 1.45
*Jussiaea linifolia *Vahl	Onagraceae	5.63 ± 1.2	4.75 ± 0.98
*Phyllanthus niruri *L.	Euphorbiaceae	4.84 ± 0.9	3.86 ± 0.74
*Hedyatis corymbosa* (L.) Lam.	Rubiaceae	2.32 ± 0.6	2.56 ± 0.22
*Mimosa pudica* L.	Fabaceae	1.98 ± 0.58	3.75 ± 1.01
*Alternanthera sessilis *(L.) R. Br. Ex DC.	Amaranthaceae	1.25 ± 0.25	1.03 ± 0.55
*Euphorbia hirta* L.	Euphorbiaceae	1.06 ± 0.29	2.58 ± 0.73
*Emilia sonchifolia *(L.) DC. Ex Wight	Asteraceae	0.96 ± 0.31	1.40 ± 0.56
Sedges			
*Cyperus rotundus* L.	Cyperaceae	8.77 ± 2.76	13.52 ± 2.47
*Fimbristylis miliacea* (L.) Vahl	Cyperaceae	6.63 ± 1.06	3.17 ± 1.30
*Cyperus sphacelatus* Rottb.	Cyperaceae	—	5.63 ± 1.51
*Cyperus aromaticus *	Cyperaceae	3.91 ± 0.65	0.98 ± 0.28
Grasses			
*Eleusine indica* (L.) Gaertn.	Poaceae	7.88 ± 3.10	7.31 ± 2.11
*Leptochloa chinensis* (L.) Nees	Poaceae	6.11 ± 1.42	9.21 ± 2.44
*Echinochloa colona *(L.) Link	Poaceae	2.43 ± 0.35	4.56 ± 1.02
*Digitaria ciliaris *(Retz.) Koel	Poaceae	2.67 ± 0.80	—
*Axonopus compressus* (Sw.) Beauv	Poaceae	0.32 ± 0.10	0.53 ± 0.12

**Table 3:** Weed control rating and crop toxicity rating of different herbicides.

Herbicide	Weed control rating			Phytotoxicity rating		
	Days after application			Days after application		
	7	14	21	7	14	21
Pretilachlor/safener	1	2	3	2	1	1
Propanil/thiobencarb	3	2	2	1	1	1
Cyhalofop-butyl + bensulfuron	3	2	1	1	1	1
Bispyribac-sodium	3	2	1	1	2	1
Bentazon/MCPA	4	3	1	2	2	1

DAA: Days after application of herbicide; weed control rating: 1 = excellent/satisfactory, 2 = good, 3 = fair, 4 = poor, and 5 = no/very poor control; phytotoxicity rating: 1 = very slight injury, 2 = slight injury, 3 = phytotoxic, 4 = severely phytotoxic, and 5 = crop 100% killed.

**Table 4:** Weed dry weight and weed density at different growth stages of rice variety AERON 1 as influenced by weed control treatments (averaged over seasons).

Treatment	Weed dry weight (g/m^2^)			Weed density (no./m^2^)		
	10 DAS	30 DAS	75 DAS	10 DAS	30 DAS	75 DAS
T1	2.25^b^	61.18^b,c^	52.89^b,c^	56.48^b^	122.74^b,c^	124.89^b,c^
T2	1.87^b^	63.40^b^	28.66^e,f^	46.83^b^	127.04^b^	98.66^e,f^
T3	2.43^b^	10.17^f,g^	39.68^d,e^	60.77^b^	20.38^f^	109.68^d,e^
T4	2.02^b^	11.37^f^	11.63^g,j^	50.65^b^	22.78^f^	81.63^g,h^
T5	1.80^b^	12.22^f^	6.77^i,j^	44.93^b^	24.41^f^	76.77^h^
T6	6.62^a^	47.78^d,e^	56.65^b,c^	165.40^a^	95.45^d,e^	126.65^b,c^
T7	6.53^a^	44.59^d,e^	21.87^f,g^	163.18^a^	89.01^d,e^	91.87^f,g^
T8	5.95^a^	51.20^c,d^	11.98^g,j^	148.87^a^	102.17^d^	81.98^g,h^
T9	5.92^a^	38.73^e^	48.72^c,d^	148.05^a^	77.34^e^	118.72^c,d^
T10	6.55^a^	42.43^de^	18.73^f,i^	163.45^a^	84.94^d,e^	88.73^f,h^
T11	6.42^a^	44.43^d,e^	7.87^h,j^	160.68^a^	88.94^d,e^	77.87^h^
T12	6.57^a^	50.42^d^	64.58^b^	164.18^a^	100.63^d^	134.58^b^
T13	5.93^a^	52.53^c,d^	19.37^f,h^	148.25^a^	104.80^c,d^	89.38^f,h^
T14	6.47^a^	52.62^c,d^	10.36^g,j^	161.88^a^	105.13^c,d^	80.36^g,h^
T15	0.00^c^	00.00^g^	00.00^j^	00.00^c^	00.00^g^	00.00^i^
T16	6.63^a^	105.22^a^	328.51^a^	165.83^a^	209.44^a^	299.50^a^
LSD	0.74	10.37	12.59	18.36	20.19	13.05

T1: pretilachlor/safener fb bentazon/MCPA; T2: pretilachlor/safener fb manual weeding; T3: pretilachlor/safener fb propanil/thiobencarb; T4: pretilachlor/safener fb propanil/thiobencarb fb bentazon/MCPA; T5: pretilachlor/safener fb propanil/thiobencarb fb manual weeding; T6: propanil/thiobencarb; T7: propanil/thiobencarb fb bentazon/MCPA; T8: propanil/thiobencarb fb manual weeding; T9: cyhalofop-butyl + bensulfuron; T10: cyhalofop-butyl + bensulfuron fb bentazon/MCPA; T11: cyhalofop-butyl + bensulfuron fb manual weeding; T12: bispyribac-sodium; T13: bispyribac-sodium fb bentazon/MCPA; T14: bispyribac-sodium fb manual weeding; T15: season long weed-free by manual weeding; T16: season long weedy. LSD: least significant difference.

Within a column, means sharing same alphabets are not significantly different at *P* = 0.05 probability level according to the least significant difference (LSD) test.

**Table 5:** Yield attributes, yield and relative yield loss of rice variety AERON 1 as influenced by weed control treatments (averaged over seasons).

Treatment	Panicles/ m^2^ (no.)	Filled grains/ panicle (no.)	Thousand-seed weight (g)	Grain yield (t/ha)	Relative yield loss (%)
T1	236.00^e^	57.83^e,f^	26.23^e^	4.05^e^	13.46
T2	235.17^e^	59.00^d,e^	26.28^e^	4.13d^e^	11.75
T3	244.00^d,e^	59.67^c,e^	26.90^d^	4.23^c,e^	9.62
T4	270.83^a^	63.90^a,b^	27.33^b,c^	4.49^a,c^	4.06
T5	273.50^a^	63.83^a,b^	27.40^a,b^	4.55^a,b^	2.78
T6	220.83^f^	54.17^f,g^	26.05^e^	3.67^f^	21.58
T7	254.67^c,d^	63.17^a,c^	27.27^b,c^	4.40^a,d^	5.98
T8	258.00^b,c^	62.17^a,d^	27.33^b,c^	4.43^a,c^	5.34
T9	220.50^f^	51.33^g^	26.27^e^	3.77^f^	20.44
T10	268.50^a,b^	64.12^a,b^	27.32^b,c^	4.47^a,c^	4.49
T11	273.00^a^	64.33^a,b^	27.50^a,b^	4.53^a,b^	3.21
T12	215.83^f^	53.83^f,g^	26.27^e^	3.60^f^	23.08
T13	244.17^d,e^	61.33^b,e^	26.90^d^	4.36^b,d^	6.84
T14	271.17^a^	64.50^a,b^	27.32^b,c^	4.45^a,c^	4.91
T15	275.33^a^	66.00^a^	27.62^a^	4.68^a^	00.00
T16	180.43^g^	45.83^h^	25.53^f^	1.77^g^	62.18
LSD	10.98	4.04	0.26	0.28	—

T1: pretilachlor/safener fb bentazon/MCPA; T2: pretilachlor/safener fb manual weeding; T3: pretilachlor/safener fb propanil/thiobencarb; T4: pretilachlor/safener fb propanil/thiobencarb fb bentazon/MCPA; T5: pretilachlor/safener fb propanil/thiobencarb fb manual weeding; T6: propanil/thiobencarb; T7: propanil/thiobencarb fb bentazon/MCPA; T8: propanil/thiobencarb fb manual weeding; T9: cyhalofop-butyl + bensulfuron; T10: cyhalofop-butyl + bensulfuron fb bentazon/MCPA; T11: cyhalofop-butyl + bensulfuron fb manual weeding; T12: bispyribac-sodium; T13: bispyribac-sodium fb bentazon/MCPA; T14: bispyribac-sodium fb manual weeding; T15: season long weed-free by manual weeding; T16: season long weedy. LSD: least significant difference.

Within a column, means sharing same alphabets are not significantly different at *P* = 0.05 probability level according to the least significant difference (LSD) test.

**Table 6:** Cost effectiveness of different herbicide treatments (averaged over seasons).

Ttreatment	Herbicide cost (RM/ha)	Laborer cost for spraying/ weeding (RM/ha)	Total cost (RM/ha)	Gross income (RM/ha)	Net benefit (RM/ha)
T1	117 + 60	50 + 50	277	4050	3773
T2	117	50 + 1250	1417	4130	2713
T3	117 + 252	50 + 50	469	4230	3761
T4	117 + 252 + 60	50 + 50 + 50	579	4490	3911
T5	117 + 252	50 + 50 + 1250	1719	4550	2831
T6	252	50	302	3670	3368
T7	252 + 60	50 + 50	412	4400	3988
T8	252	50 + 1250	1552	4430	2878
T9	110 + 114	50	274	3770	3496
T10	110 + 114 + 60	50 + 50	384	4470	4086
T11	110 + 114	50 + 1250	1524	4530	3006
T12	120	50	170	3600	3430
T13	120 + 60	50 + 50	280	4360	4080
T14	120	50 + 1250	1420	4450	3030
T15	0.0	2500	2500	4680	2180
T16	0.0	0.0	0.0	1770	1770

T1: pretilachlor/safener fb bentazon/MCPA; T2: pretilachlor/safener fb manual weeding; T3: pretilachlor/safener fb propanil/thiobencarb; T4: pretilachlor/safener fb propanil/thiobencarb fb bentazon/MCPA; T5: pretilachlor/safener fb propanil/thiobencarb fb manual weeding; T6: propanil/thiobencarb; T7: propanil/thiobencarb fb bentazon/MCPA; T8: propanil/thiobencarb fb manual weeding; T9: cyhalofop-butyl + bensulfuron; T10: cyhalofop-butyl + bensulfuron fb bentazon/MCPA; T11: cyhalofop-butyl + bensulfuron fb manual weeding; T12: bispyribac-sodium; T13: bispyribac-sodium fb bentazon/MCPA; T14: bispyribac-sodium fb manual weeding; T15: season long weed-free by Manual weeding; T16: season long weedy.

RM: Ringgit Malaysia. Market price of herbicide commercial products: pretilachlor/safener (Soffit N300 EC) = 70 RM/L, cyhalofop-butyl (Halop 100 EC) = 110 RM/L, bensulfuron (Tekong) = 19 RM 100/g, bispyribac-sodium (Nominee 100 SC) = 98 RM 250/mL, propanil/thiobencarb (Satuni) = 42 RM/L, and bentazon/MCPA (basagran M60) = 38 RM/L.

Manual weeding cost: 100 laborers/ha for 2 weedings at 25 RM/laborer/day, herbicide application cost: 2/laborer/ha/round at 25 RM/laborer/day, market price of paddy: 1000.00 RM t/ha, gross income = paddy yield (t/ha) × market price (RM t/ha), and net benefit = gross income − total weeding cost.

1 US$ = 3 RM (approx.).
